# Gcn5 and Rpd3 have a limited role in the regulation of cell cycle transcripts during the G1 and S phases in *Saccharomyces cerevisiae*

**DOI:** 10.1038/s41598-019-47170-z

**Published:** 2019-07-23

**Authors:** A. Kishkevich, S. L. Cooke, M. R. A. Harris, R. A. M. de Bruin

**Affiliations:** 1 MRC Laboratory for Molecular Cell Biology University College London, WC1E 6BT London, UK; 2 UCL Cancer Institute, University College London, WC1E 6BT London, UK; 3 Present Address: Department of Biochemistry, University of Oxford, OX3 1QU Oxford, UK

**Keywords:** Transcription, Cell-cycle exit

## Abstract

Activation of cell cycle regulated transcription during the G1-to-S transition initiates S phase entry and cell cycle commitment. The molecular mechanisms involving G1/S transcriptional regulation are well established and have been shown to be evolutionary conserved from yeast to humans. Previous work has suggested that changes to the chromatin state, specifically through histone acetylation, has an important role in the regulation of G1/S transcription in both yeast and human cells. Here we investigate the role of histone acetylation in G1/S transcriptional regulation in the budding yeast *Saccharomyces cerevisiae*. Our work shows that histone acetylation at specific sites at G1/S target gene promoters peaks at the G1-to-S transition, coinciding with their peak transcription levels. Acetylation at G1/S target promoters is significantly reduced upon deletion of the previously implicated histone acetyltransferase Gcn5, but G1/S cell cycle regulated transcription is largely unaffected. The histone deacetylase Rpd3, suggested to have a role in Whi5-dependent repression, is required for full repression of G1/S target genes in the G1 and S phases. However, in the context of transcriptionally active levels during the G1-to-S transition, this seems to play a minor role in the regulation of cell cycle transcription. Our data suggests that histone acetylation might modulate the amplitude of G1/S cell cycle regulated transcription in *Saccharomyces cerevisiae*, but has a limited role in its overall regulation.

## Introduction

Cell cycle regulated transcription plays an important role in cell cycle control; in most eukaryotic cells there are three main transcriptional waves associated with distinct stages of the cell cycle: G1/S, G2/M and M/G1^1^. Activation of G1/S transcription is required for the initiation of DNA replication, which marks S phase entry and cell cycle commitment^2^. A large body of work has shown that the molecular mechanisms involved in both the activation and inactivation of G1/S transcription are conserved from yeast to humans. In short G1/S transcription factor complexes are kept inactive in G1 by the binding of transcriptional inhibitors. Accumulation of Cyclin Dependent Kinase (CDK) activity results in inactivation of the inhibitors and activation of G1/S transcription, whilst inactivation of this transcriptional wave is largely achieved through the accumulation of transcriptional repressors, which are G1/S targets themselves^2^.

In addition to the well-established role for the G1/S transcription factor complexes, changes to the chromatin state via histone modifications at G1/S target gene promoters are also thought to be important in the regulation of G1/S transcription. Of these, histone acetylation in particular has been linked to G1/S transcriptional regulation. Generally, acetylation of histones is thought to open chromatin and is linked to active transcription, whilst deacetylation is associated with closed chromatin and transcriptional repression (reviewed in^3^). Indeed, numerous studies have associated histone acetylation with actively transcribed genes in both yeast^4–7^ and higher eukaryotes^8^.

In human cells the requirement of histone acetylation for G1/S transcriptional regulation has been suggested by a number of studies^9–11^. For example the histone acetyltransferase (HAT) Gcn5 has been linked to the regulation of G1/S transcription and is required for accurate G1-to-S transition^11^. In addition, it has been suggested that repression of G1/S transcription in G1 phase is dependent on recruitment of the histone deacetylase (HDAC) HDAC1 to G1/S target promoters by the transcriptional inhibitor Rb^12–14^.

In the budding yeast, *Saccharomyces cerevisiae*, G1/S transcription is regulated by two distinct transcription factor complexes: SBF and MBF^15–21^. Whilst SBF and MBF target genes have similar temporal patterns of expression, the mechanisms of their regulation are distinct. SBF is a transcriptional activator whose activity is inhibited by Whi5 in early G1 phase^21,22^, whilst MBF is a transcriptional repressor and acts together with its co-repressor Nrm1 outside of G1 phase^23^. The role of these transcriptional regulators is well established and it has been suggested that histone acetylation plays an important part in how these complexes control G1/S transcription. Similar to the case in human cells, in budding yeast Rpd3, the homologue of HDAC1, has been implicated in the regulation of G1/S targets via interaction with the transcriptional repressor Whi5^5,24,25^. Rpd3 is the catalytic subunit of two HDAC complexes; Rpd3S and Rpd3L^26^. It is recruited to SBF target promoters in asynchronous cells^5^ as well as in G1 phase^24^. Moreover, transcript levels of the SBF targets *SVS1* and *CLN2* are upregulated in an asynchronous *rpd3*∆ culture^27^. In addition, Gcn5, the yeast HAT subunit of the transcription co-activator complex SAGA^28,29^ has been shown to bind both SBF and MBF target genes^5,30^.

Based on these studies it has been suggested that histone acetylation plays an important role in the regulation of G1/S transcription. However, how the recruitment of HATs and HDACs affect the histone acetylation state at G1/S target promoters during the cell cycle and how it contributes to the regulation of G1/S transcription remains largely untested. Here we investigate the role of histone acetylation in the regulation of G1/S transcription in the budding yeast, *Saccharomyces cerevisiae*. In line with the published data on the recruitment of both Rpd3 and Gcn5 to G1/S target promoters, our data shows that histone acetylation at G1/S target promoters is cell cycle regulated and largely depends on Gcn5. However, our data suggest that these changes to the histone acetylation state at G1/S target promoters have a limited role in the regulation of G1/S cell cycle transcription.

## Results

### Histone acetylation at G1/S target promoters is cell cycle-regulated

We wanted to establish if, in budding yeast, histone acetylation levels at G1/S target promoters change during the cell cycle and how this correlates with G1/S transcript levels. We focused on acetylation of histone H3 Lysine 9 and Lysine 14 (H3K9ac and H3K14ac), known residues acetylated by the HAT Gcn5^31^, previously shown to be recruited to G1/S target promoters^5^. Exponentially growing wild-type cells were synchronised by the mating pheromone α-factor and samples were collected every 15 min after release until 75 min and processed for RNA extraction and Chromatin Immunoprecipitation (ChIP) analysis. Cell cycle synchrony and progression was monitored by budding index (Supplementary Fig. 1). The budding index increases at 45 min, reaching maximum levels at 60 minutes, indicative of cells entering S phase 30 minutes after release.

**Figure 1:**
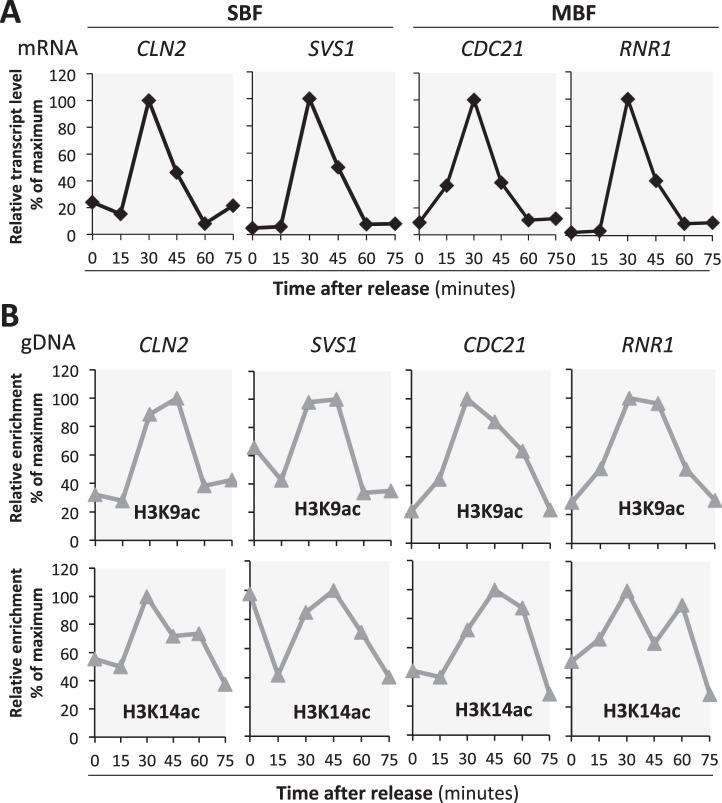
Acetylation of histone H3 lysines 9 and 14 at G1/S target promoters is cell cycle regulated. Exponentially growing wild-type yeast cultures were arrested in G1 phase with α-factor and then released into the cell cycle. Samples were collected for Chromatin Immunoprecipitation (ChIP) and transcription analysis. (**A**) Transcript levels of SBF targets *CLN2* and *SVS1* and MBF targets *CDC21* and *RNR1* were measured by RT-qPCR and normalized to *ACT1* levels. Levels relative to the maximum value (100%) are plotted against time from release. (**B**) Relative enrichment of the same genes measured by ChIP with antibodies specific to H3K9ac and H3K14ac. Signal was normalized to total H3 ChIP and signal at *ACT1*, and levels are relative to the maximum value (100%) plotted against time from release.

Here we have investigated G1/S targets regulated by SBF (*CLN2, SVS1*) and MBF (*CDC21, RNR1*), which are well characterised G1/S targets and their regulation is thought to be representative of other genes in the G1/S regulon^32,33^. The expression levels of these G1/S target genes, measured by RT qPCR, increase at 30 minutes and are subsequently downregulated when cells enter S phase (Fig. 1A). Acetylation levels were examined by chromatin immunoprecipitation with antibodies against H3K9ac and H3K14ac. It is important to note that, based on the manufacturer’s information, the H3K9Ac antibody shows good specificity when compared to a H3K9Ac mutant control (~4 fold), but the H3K14Ac antibody appears less specific compared to the H3K14Ac mutant control (<2-fold). We observe that H3K9ac and H3K14ac at the four independent G1/S target gene promoters oscillate during the cell cycle with maximum levels 30–45 minutes after release, corresponding with peak gene expression (Fig. 1B). These data are in line with previous work showing that histone acetylation is linked to active transcription.

### Acetylation at SBF and MBF target promoters is dependent upon Gcn5

**Figure 2:**
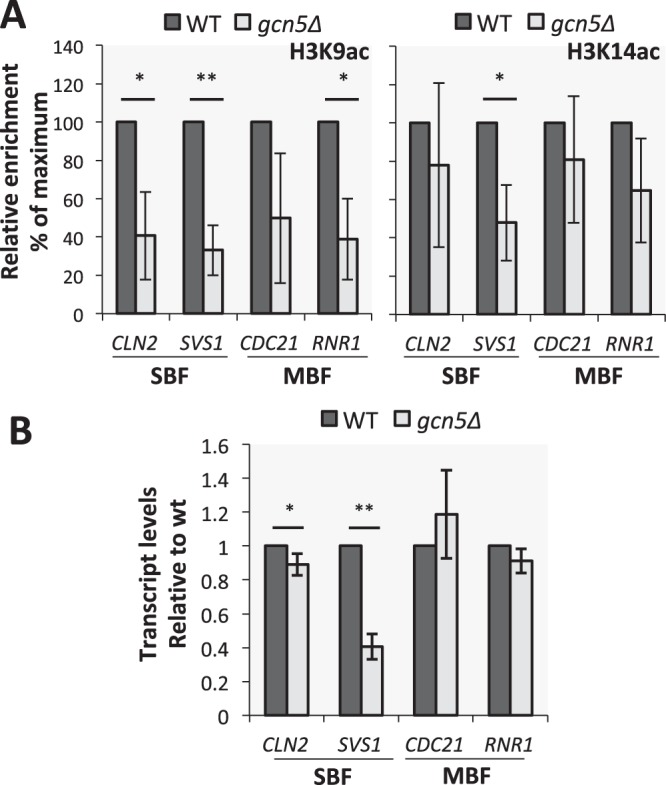
Histone acetyltransferase Gcn5 is responsible for acetylation of histone H3 lysines 9 and 14 at G1/S target promoters. Chromatin Immunoprecipitation and transcriptional analysis of exponentially growing asynchronous wild-type and *gcn5*∆ yeast cultures. (**A**) Relative enrichment measured at indicated gene promoters by immunoprecipitation using antibodies specific to H3K9ac and H3K14ac in wild-type (dark grey) and *gcn5∆* (light grey) cells. Signal was normalized to total H3 ChIP and enrichment at *S. pombe ACT1*. Levels are presented relative to wild-type (100%). Error bars represent standard deviation, n = 4. (**B**) Transcript levels of SBF targets, *CLN2* and *SVS1*, and MBF targets, *CDC21* and *RNR1*, in wild-type and *gcn5∆* cells, measured by RT-qPCR. Values are normalized to *ACT1* signal and relative to wild-type (1.0). Error bars represent standard deviation, n = 4. *p-value ≤ 0.05, **p-value ≤ 0.01, other results are non-significant.

Next we wanted to test if H3K9ac and H3K14ac at G1/S target promoters depend on Gcn5. We tested H3K9ac and H3K14ac by ChIP in asynchronous wild-type and *gcn5*∆ cells. As *gcn5*∆ is known to have reduced global acetylation at these residues^34,35^, we did a spike-in with *S. pombe* cells to normalise our ChIP results to the *S. pombe ACT1* promoter, for a more accurate comparison between the WT and *gcn5*∆ samples. Our data shows that H3K9ac and H3K14ac at G1/S target promoters is reduced in *gcn5*∆ cells in comparison to wild-type cells at the tested promoters. Notably there is a significant decrease in both H3K9ac and H3K14ac at the *SVS1* promoter, and in H3K9ac at the *CLN2* and *RNR1* promoters (Fig. 2A). Deletion of *GCN5* results in a significant decrease in transcript levels of *CLN2* and *SVS1*, with no significant changes in expression of *CDC21* and *RNR1* (Fig. 2B). These data show that histone acetylation at G1/S target promoters largely depends on Gcn5, however this does not always correspond to decreased transcript levels in an asynchronous population.

### Gcn5 is not required for activation of G1/S transcription

To establish the role of Gcn5 in regulating G1/S transcription we decided to monitor cell cycle-regulated transcription in synchronized populations of wild-type and *gcn5*∆ cells. Interestingly, *gcn5*∆ cells proved difficult to arrest by the mating pheromone α-factor (Supplementary Fig. 2A) requiring a higher concentration and longer exposure to α-factor. This is unexpected if Gcn5 is involved in the activation of G1/S transcription since an α-factor arrest requires keeping G1/S transcription inactive. However, Gcn5 is thought to have a role in global gene expression^34,36^ so inactivation could compromise the cells ability to arrest by affecting the mating transcriptional programme. To test this we monitored the expression levels of the well established mating transcriptional programme targets *FUS3* and *FAR1*^37^ during an α-factor arrest. Our data shows that these genes are activated to similar levels in cells arrested by α-factor in both wild-type and *gcn5∆* cells (Supplementary Fig. 3). This suggests that the impaired ability of *gcn5*∆ cells to arrest by α-factor is unlikely to be caused by reduced transcription of mating genes.

**Figure 3:**
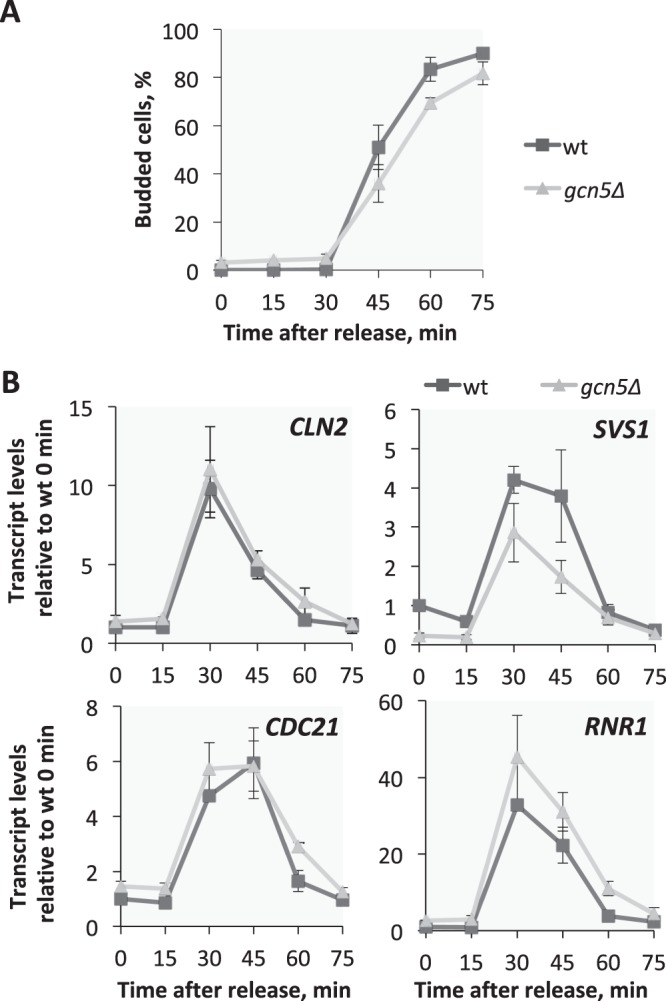
G1/S transcription is not deregulated in cells lacking Gcn5. Cell cycle regulated transcription in synchronized wild-type and *gcn5∆* cells. (**A**) Budding index as percentage of budding cells. (**B**) Transcript levels of SBF targets, *CLN2* and *SVS1*, and MBF targets, *CDC21* and *RNR1*, measured by RT-qPCR at indicated time-points after release from G1 arrest, in wild-type (dark grey) and *gcn5∆* (light grey) cells. Relative fold induction, over wild-type time point 0, is plotted. Error bars represent standard deviation, n = 3.

Wild-type and *gcn5*∆ cells were synchronised using a higher concentration and longer exposure to α-factor. It is important to note that whilst *gcn5*∆ cells do arrest well in these conditions a small fraction of *gcn5*∆ cells remain in G1 after release (Supplementary Fig. 3A, B). In addition, we observed a minor cell cycle delay in the *gcn5*∆ cells, as determined by budding index (Fig. 3A) and flow cytometry (Supplementary Fig. 2B), which has been reported previously^38,39^. This minor cell cycle effect is important to take into account when interpreting expression levels during the cell cycle and in asynchronous cell populations.

The strains were analysed for histone acetylation and transcription after α-factor arrest and release. A reduction in H3K9ac and H3K14ac was observed at the G1/S target genes in *gcn5*∆ compared to wild-type cells at all time points (Supplementary Fig. 4), similarly to our observations in an asynchronous population. The transcriptional analysis shows that both SBF and MBF G1/S targets are still cell cycle regulated in the absence of Gcn5 (Fig. 3B). Only the SBF target *SVS1* shows a slight reduction in peak transcription, but the SBF target *CLN2* and the MBF targets *RNR1* and *CDC21*, reach similar peak transcript levels to wild-type. These data indicate that cell cycle regulated transcription is largely unaffected in the absence of Gcn5, suggesting a limited role for Gcn5-dependent histone acetylation at G1/S target promoters in the regulation of G1/S transcription.

### Histone deacetylase Rpd3 is required for full repression outside of the G1-to-S transition

**Figure 4:**
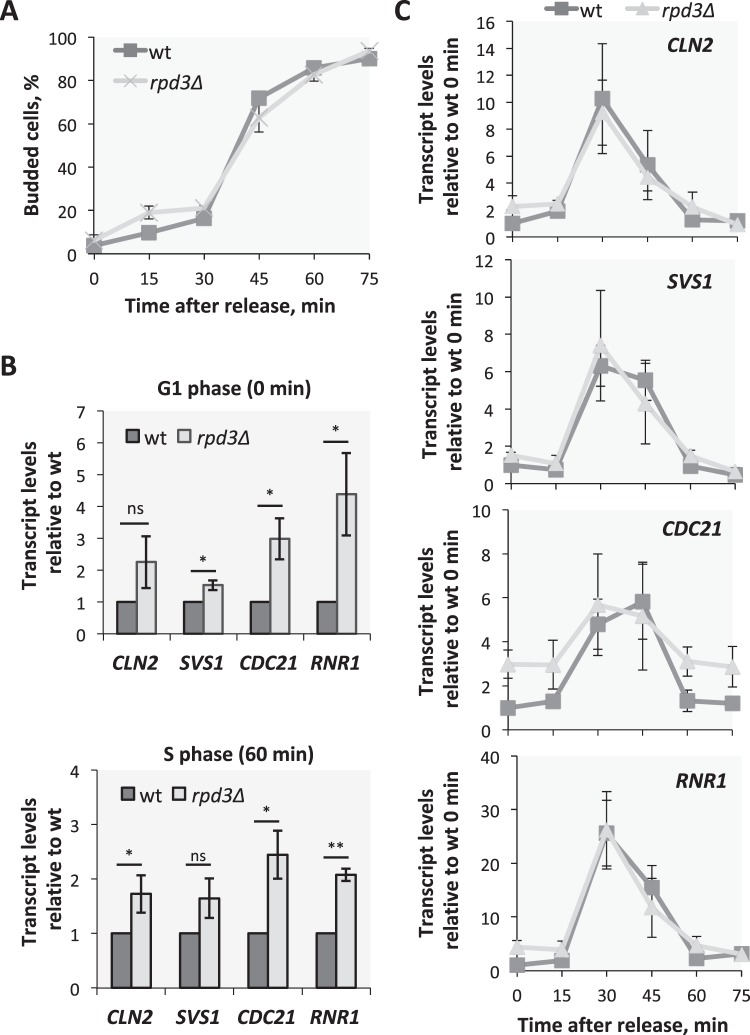
Rpd3 is involved in full repression of G1/S target genes outside the G1-to-S transition. Cell cycle regulated transcription in synchronized experiment of wild-type and *rpd3∆* cells. **(A)** Budding index as percentage of budding cells. **(B)** Transcript levels of SBF targets, *CLN2* and *SVS1*, and MBF targets, *CDC21* and *RNR1*, measured by RT-qPCR in G1 phase (0 min after release) and in S phase (60 min after release) and **(C)** during the cell cycle in wild-type (dark grey) and *rpd3∆* (light grey) cells. Relative fold induction, over wild-type **(A**, **B)** and wild-type time point 0 (**C**) is plotted. Error bars represent standard deviation, n = 3. *p-value ≤ 0.05, **p-value ≤ 0.01.

Whilst histone acetylation is associated with active transcription, histone deacetylation is linked to repression. Previous work has suggested that the HDAC Rpd3 is recruited to SBF target promoters by the transcriptional inhibitor Whi5 to repress their transcription in G1 phase^24^. However, the role of Rpd3 in transcriptional repression of G1/S targets in G1 phase has not been tested. To investigate the role of Rpd3 in regulating G1/S transcription we monitored gene expression in wild-type and *rpd3∆* cultures synchronised by α-factor. Samples for RNA extraction were collected every 15 min after release up to 75 min and progression through the cell cycle was monitored by budding index (Fig. 4A) and flow cytometry (Supplementary Fig. 5). Although cell cycle progression is comparable based on budding index, the flow cytometry analysis shows that cells lacking Rpd3 enter S phase more quickly than WT following α-factor arrest. However, apart from this small acceleration in S phase entry, based on DNA content, the synchrony of *rpd3*∆ cells is very similar to that of wild-type cells at the specific time points investigated, 0, 30 and 60 minutes.

Transcript levels of both SBF (*CLN2, SVS1*) and MBF targets (*CDC21, RNR1*) in *rpd3*∆ cells are slightly, but largely significantly, upregulated in G1 phase (Fig. 4B upper panel) and S phase (Fig. 4B lower panel) compared to wild-type cells. These results indicate that Rpd3 is required for maximum repression of G1/S transcription outside of the G1-to-S transition following arrest and release from α-factor. Indeed, up-regulation of transcripts during G1 could contribute to the accelerated S phase entry observed by flow cytometry.

However, the transcript levels observed upon de-repression of these targets in *rpd3*∆ cells during the G1 and S phases of the cell cycle are significantly lower than their transcriptionally active levels at the G1-to-S transition (30 min). Therefore, whilst Rpd3 is involved in the repression of G1/S targets, cell cycle regulated transcription is largely maintained in *rpd3*∆ cells (Fig. 4C). Together these data indicate that Rpd3 is required for full repression of G1/S targets outside of the G1-to-S transition. However, in the absence of Rpd3, G1/S transcription is still cell cycle regulated suggesting that Rpd3 modulates G1/S transcription but is not necessary for its regulation.

## Discussion

In this study we have investigated the role of histone acetylation and deacetylation in the regulation of G1/S transcription in the budding yeast, *Saccharomyces cerevisiae*. We focussed on establishing the role of the HAT, Gcn5, and HDAC, Rpd3, which have both been shown to be recruited to G1/S target gene promoters^5,24,27^. Our data establishes that acetylation of lysines 9 and 14 on histone H3 is cell cycle regulated with maximum levels coinciding with peak G1/S transcription levels. Inactivation of the HAT Gcn5 significantly decreases histone acetylation at these specific sites indicating that acetylation of lysines 9 and 14 on histone H3 at G1/S target promoters are Gcn5-dependent. However, Gcn5 inactivation does not lead to substantial changes in G1/S transcription levels, suggesting a limited role for Gcn5-dependent acetylation in G1/S transcriptional regulation.

Deletion of Rpd3, implicated in Whi5-dependent repression of G1/S transcription in G1, leads to de-repression of both SBF and MBF target genes in the G1 and S phases. Whi5 is an SBF specific transcriptional inhibitor that is inactive, through CDK-dependent phosphorylation, outside of the G1 phase of the cell cycle^21^. Based on the data presented here Rpd3-dependent repression of G1/S transcription is unlikely to be Whi5-specific. Overall, the loss of Rpd3-dependent repression in the G1 and S phases is largely insignificant when put in the context of peak transcript levels, observed during the G1-to-S transition. Based on this our data suggest that the HDAC Rpd3 has a limited role in the context of regulating cell cycle dependent transcript levels in proliferating cells. However, our data shows that Rpd3 is required for full repression of G1/S transcription in G1, which is in line with the suggested possible role for Rpd3 in achieving a reversible transition from proliferation to quiescence^40^. Initiating the transition from proliferation to quiescence in G1 requires full repression of G1/S transcription, which our data shows requires Rpd3 activity.

Although there were no major perturbations to G1/S transcription upon deletion of Gcn5 or Rpd3, both mutants exhibit altered cell cycle progression. Cells lacking Gcn5 are delayed in entering S phase upon release from α-factor arrest compared to wild-type, conversely cells lacking Rpd3 enter S phase more quickly. This suggests that these enzymes, and histone acetylation, may still play a broader role in cell cycle regulation.

We focussed our study upon Rpd3 and Gcn5 as they have both been previously implicated in G1/S transcriptional regulation. However, they may have redundancy with other HATs and HDACs, and it would be interesting to study G1/S transcriptional regulation upon further deregulation of histone acetylation. In addition our findings are limited to the context of synchronisation with α-factor. Whilst previous studies have indicated that G1/S transcriptional regulation is comparable between α-factor synchronisation and centrifugal elutriation^21,23,41^, which is thought to be more physiological, we acknowledge that it would be insightful to test our findings using alternative synchronization methods to study the G1/S transition.

Regulation of the G1/S transcriptional wave is conserved from yeast to human^2^ and is established in detail in the budding yeast *Saccharomyces cerevisiae*^15–23,42,43^. This makes budding yeast an excellent system to study regulation of G1/S transcription at the chromatin level. Histone acetylation has been implicated in regulation of G1/S transcription in both yeast and human cells^9–11,24^. No direct role of these histone-modifying enzymes in regulation of G1/S transcription has been established in yeast, and previous studies were performed in asynchronous cultures. Our results are in line with the previous studies in human cells, where depletion of the HAT Gcn5 leads to a reduction in transcript levels, indicating the Gcn5 is required for full activation of some G1/S target genes, but loss of Gcn5 does not prevent transcriptional activation nor does it induce a cell cycle arrest^11^. The same is true for the HDAC Rpd3. Rpd3 is a homolog of HDAC1 in human cells^12–14^. HDAC1 was shown to be required for full repression of G1/S transcription in G1 phase, since its depletion leads to insufficient repression. However, this does not result in a complete loss of repression nor does it have a significant effect on cell cycle progression.

Our findings support the notion that histone marks have a role in the ‘fine-tuning’ of transcription. Zhang and colleagues established that in yeast, activation of genes in heterochromatic regions do not require changes in the chromatin state. However, the transcription of highly transcribed stress response genes, artificially placed into heterochromatic regions, was reduced suggesting that histone marks affect basal levels of transcription and not transcriptional activation or inactivation^44^. Our data shows that inactivation of either Gcn5 or Rpd3 has a limited effect on cell cycle regulated transcription, regardless of whether the gene is regulated by SBF or MBF. Our study provides another piece of evidence to support the hypothesis that whilst transcription factors are responsible for transcriptional regulation, histone acetylation influences the basal level of transcription, which could contribute to the modulation of a transcriptional response and have an important role when cells are exposed to specific conditions.

## Materials and Methods

### Yeast strains and media

The following yeast strains were used in this study: wild-type (*15Daub*: *MATa, ade1, leu2-3, 112 his2, trp1-1, ura3∆ns, bar1∆*); *rpd3∆* (*15 Daub rpd3::URA3)* and *gcn5∆* (*15 Daub gcn5::KAN)*. Strains were derived from wild-type *15 Daub* using PCR-based method (Longtine *et al*., 1998). All cultures were grown in YPD (Formedium CCM0205) at 30 °C with aeration.

For spike-in of *S. pombe* for ChIP the wild-type (*h* + *leu1-32 ura4-D18*) strain was grown in YES broth (Formedium PCM0310) at 30 °C with aeration.

### Cell cycle synchronisation with mating pheromone

8 µl α-factor (2 mg/ml stock concentration) was added into 100 ml of exponentially growing yeast cultures OD_600_ = 0.6–0.8. Cultures were incubated for at least 90 min and efficiency of synchronisation was monitored by number of budding cells under Zeiss light microscope. For the *gcn5*∆ time-courses with *gcn5*∆ cells were arrested in 1.2 µg/ml α-factor for 3 hours, due to difficulty in arresting the *gcn5*∆ mutant. The cultures were then washed with fresh media and released into warm fresh rich media for the rest of the time course.

### RNA extraction and RT-qPCR

25 ml of yeast culture was collected for RNA extraction at each time point. Cells were pelleted and snap frozen at −80 °C. RNA extraction was performed with RNeasy Plus Mini Kit (Qiagen 74134) according to the manufacture’s protocol. Before extraction the pellet was disrupted with glass beads (Biospec 11079105) in RLT buffer supplemented with 1% of β-mercaptoethanol. Total RNA was diluted to 20 ng/µl and 4 µl was used for 14 µl qPCR-reaction using One step qRT-PCR MasterMix for SYBR® assay no ROX (Eurogentec SYRT-032XNR) with Euroscipt/RNase inhibitor. Reactions were run on Chromo-4 Real-Time PCR detector (Bio-Rad). Obtained data was processed by Bio-Rad CFX Manager 3.0 software. The data was normalised against actin and analysed using the Ct value method. Statistical significance was tested with a ratio paired t-test on values before normalisation to wild-type using Graphpad prism software.

### Chromatin immunoprecipitation and qPCR

Analysis of histone modifications were performed by Chromatin Immunoprecipitation (ChIP) combined with quantitative PCR. Cross-linking was performed with 45 ml of culture and 1% formaldehyde for 20 min at room temperature. The reaction was terminated with 2.5 M Glycine for 5 min, cells were spun down and the pellet was washed twice with ice-cold TBS (Tris-HCl 50 mM/NaCl 150 mM, pH 7.5). The pellet was suspended in 5 ml of TBS and 1 ml aliquots were frozen in liquid nitrogen. The cells were disrupted in 500 µl ice-cold ChIP lysis buffer (HEPES-KOH pH 7.5 50 mM/NaCl 140 mM/Triton X-100 1%/Sodium Deoxycholate 0.1%/EDTA 1 mM) supplemented with Protease Inhibitors (Roche 04693124001) by shaking with glass beads (BioSpec Products 11079105) for 20 min. Chromatin pellet was suspended in 500 µl of ChIP lysis buffer supplemented with protease inhibitors. DNA was sheared by sonication (Qsonica sonicator, amplitude 100%, process time 5 min with pulse-ON 30 sec and pulse-OFF 2 min). 5 µl of the lysate was aliquoted into separate tube for whole cell extract. Antibodies H3K9ac (Millipore 07-352), H3K14ac (Millipore 07-353) and H3 (Abcam ab1791) were added according to manufacturer’s instructions and samples were incubated at 4 °C overnight rotating. The lysates were incubated with 35 µl of 50% suspension of Protein A-Sepharose beads (Sigma P3391-1.5 G) rotating for 3 hours and then washed 6 times with 1 ml of freshly prepared cold wash buffer (Tris-HCl pH 7.5/Triton X-100 1%/NaCl 150 mM/EDTA 5 mM/NP-40 0.5%). To de-cross link beads were mixed with 100 µl of 10% Chelex Resin (Bio-Rad 142-1253). Chelex suspension was also added to corresponding whole cell extract. The mixture was shaken for 10 seconds, boiled for 10 min and then spun for 1 min at 12,000 rpm. Then 70 µl of the supernatant was moved to a new tube. 120 µl of water (Millipore H2OMB0501) was added to the remaining Chelex. The mixture was shaken and spun again. 100 µl of the supernatant were added to the previous 70 µl. DNA samples were used to run qPCR on Chromo-4 Real-Time PCR detector (Bio-Rad). The reactions were run in 14 µl with One step qRT-PCR MasterMix for SYBR® assay No ROX (Eurogentec SYRT-032XNR) without Euroscript. Obtained data was processed by Bio-Rad CFX Manager 3.0 software. Data was analysed as percentage of the whole cell extract and values normalised to Histone H3 occupancy. In wild-type cell cycle synchrony experiments enrichment was normalised to levels at the *ACT1* promoter and then calculated as percentage of wild-type maximum enrichment. Statistical significance was tested with a ratio paired t-test on values before normalisation to wild-type using Graphpad prism software.

In order to compare histone acetylation between wild-type and the *gcn5*∆ mutant, *S. pombe* cells were fixed together with the *S. cerevisiae* strains. 4 ml of *S. pombe* at the same OD was added to 40 ml *S. cerevisiae* culture in a final concentration of 1% formaldehyde. The subsequent ChIP steps were carried out as above, and the data normalised to the *ACT1* promoter of *S. pombe* instead of *S. cerevisiae*.

### DNA content analysis by flow cytometry

The efficiency of the arrest in G1 phase with mating pheromone was assessed by flow cytometry. For this wild-type and *gcn5*∆ exponentially growing cultures were arrested as described above. 500 µl of yeast culture was mixed with cold 1 ml of 90% ethanol and left at −20 °C for at least 18 hours. Fixed cells were pelleted at 5000 g for 20 min at 4 °C. The pellet was then washed by mixing with 800 µl of 50 mM sodium citrate buffer pH 7.2, left at room temperature for 10 min and spun at 5000 g for 5 min at room temperature twice. Then cells were suspended in 500 µl of 50 mM sodium citrate pH 7.2 containing 20 µg/ml RNase A (Sigma, R4875) and 2.5 µM Sytox Green (Thermo Scientific, S7020). After at least 1 hour incubation at 37 °C in the dark 20 µL of 10 mg/ml proteinase K (Sigma, P2308) was added and the mixture was incubated at 55 °C in the dark for at least an hour. Then samples were stored at 4 °C in the dark until being further processed. Before analysis by flow cytometry samples were sonicated using microtip Branson Sonifier (output 5; 30 sec pulses). DNA content of 50,000 cells was analysed using BD LSR II Flow Cytometer.

## Supplementary information

Supplementary Figure 1-5
