# Supplementary material for: Gcn5 and Rpd3 have a limited role in the regulation of cell cycle transcripts during the G1 and S phases in Saccharomyces cerevisiae
Source: Sci Rep. 2019 Jul 23;9:10686. doi: 10.1038/s41598-019-47170-z (PMC6650506; doi:10.1038/s41598-019-47170-z)
Supplement: Supplementary file 1 — Supplementary Figure 1-5 [file 41598_2019_47170_MOESM1_ESM.pdf]

Gcn5 and Rpd3 have a limited role in the regulation of cell cycle transcripts during the G1 and S phases in *Saccharomyces cerevisiae*.

Kishkevich, A., Cooke, S.L., Harris, M.R.A. and de Bruin, R.A.M

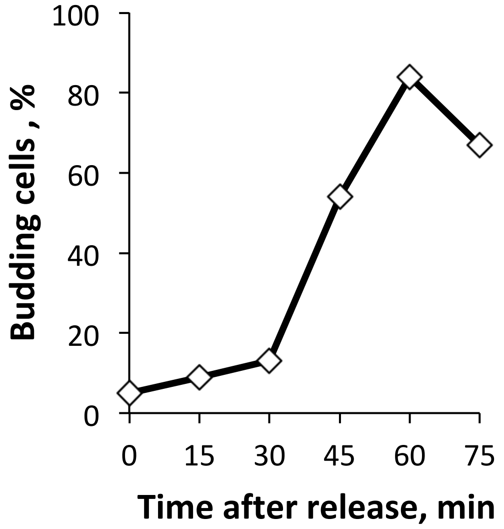

Supplementary Figure 1. Budding index in wild-type cells for cell cycle synchrony experiment. Budding index, as percentage of budded cells, in synchronized experiment of wild-type cells for data shown in figure 1.

Gcn5 and Rpd3 have a limited role in the regulation of cell cycle transcripts during the G1 and S phases in *Saccharomyces cerevisiae*.

Kishkevich, A., Cooke, S.L., Harris, M.R.A. and de Bruin, R.A.M

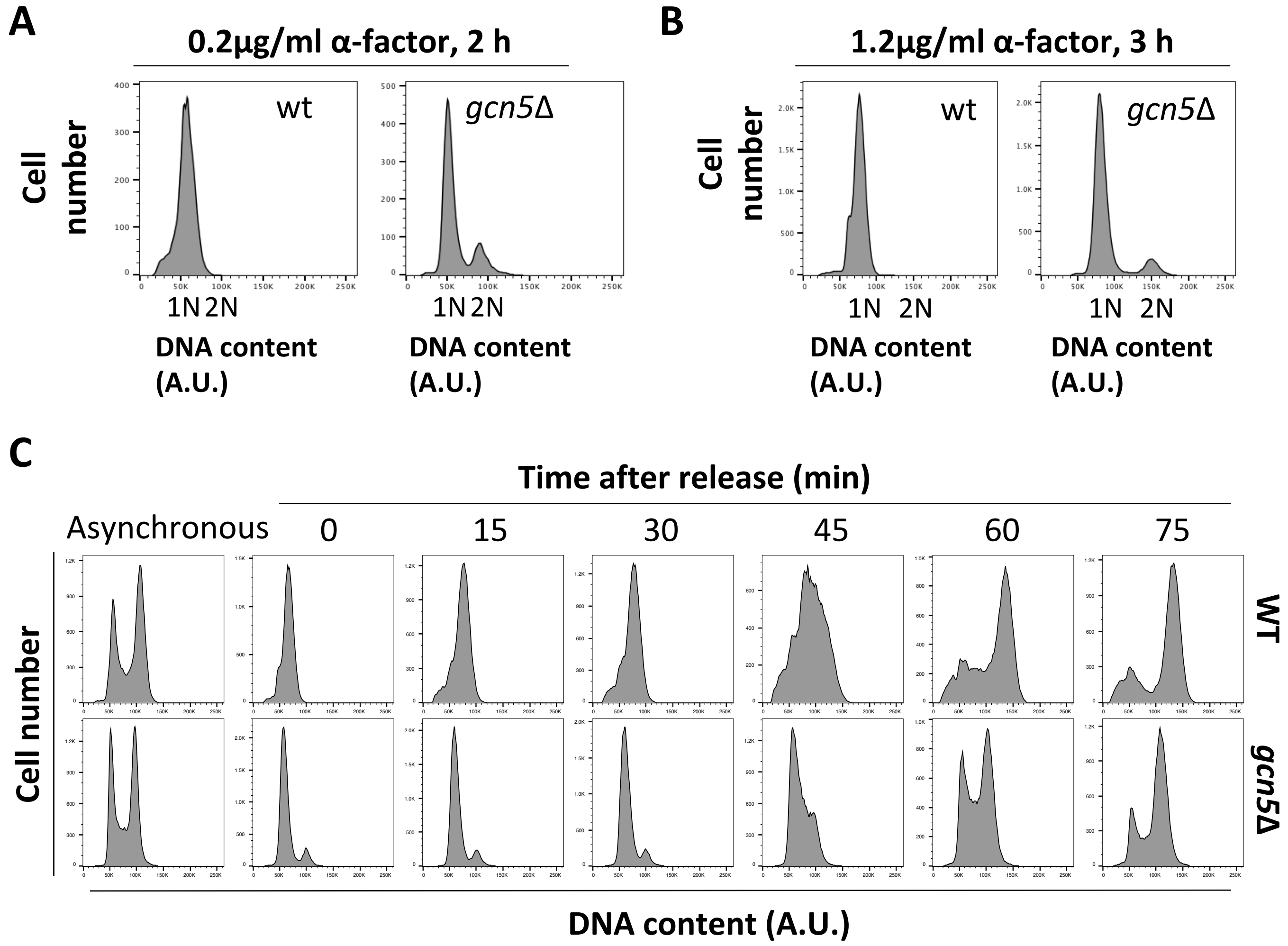

Supplementary Figure 2. Cells lacking Gcn5 do not arrest effectively by the mating pheromone α-factor and have a minor cell cycle delay when released. α-factor cell cycle arrest of wild-type and *gcn5Δ* cell cultures. **(A)** FACS analysis of wild-type and *gcn5Δ* cells arrested by α-factor under standard conditions and **(B)** increased concentration and time of exposure. **C)** FACS analysis of α-factor arrested and released wild-type and *gcn5Δ* cells cultures. Plot is representative of three independent experiments. The spread in DNA staining seen in wild-type cells is likely due to the large variation in cell size resulting from prolonged exposure to α-factor.

Gcn5 and Rpd3 have a limited role in the regulation of cell cycle transcripts during the G1 and S phases in *Saccharomyces cerevisiae*.

Kishkevich, A., Cooke, S.L., Harris, M.R.A. and de Bruin, R.A.M

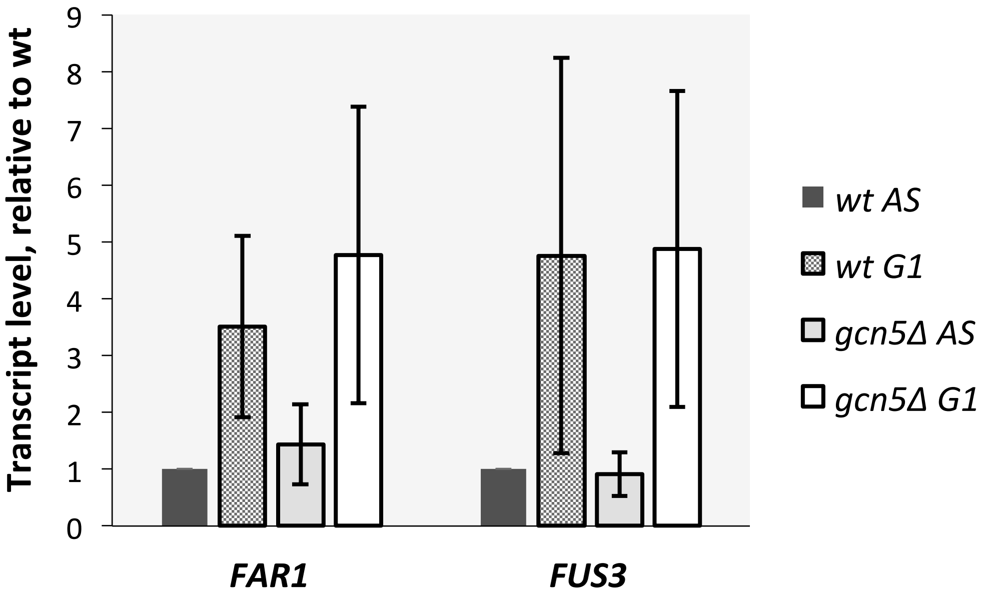

Supplementary Figure 3. Gcn5 is not required for activation of the mating transcription program. Exponentially growing wild-type and *gcn5Δ* cultures were treated with the same concentration of α-factor for 2 hours. Transcript levels of mating program genes *FAR1* and *FUS3* were measured by RT-qPCR in wild-type asynchronous (dark grey), wild-type G1 arrested (grey patterned), *gcn5Δ* asynchronous (light grey) and *gcn5Δ* G1 arrested (white) cultures and normalised against *ACT1* levels. Transcript levels are fold increase relative to wild-type asynchronous, error bars represent standard deviation, n = 3.

Gcn5 and Rpd3 have a limited role in the regulation of cell cycle transcripts during the G1 and S phases in *Saccharomyces cerevisiae*.

Kishkevich, A., Cooke, S.L., Harris, M.R.A. and de Bruin, R.A.M

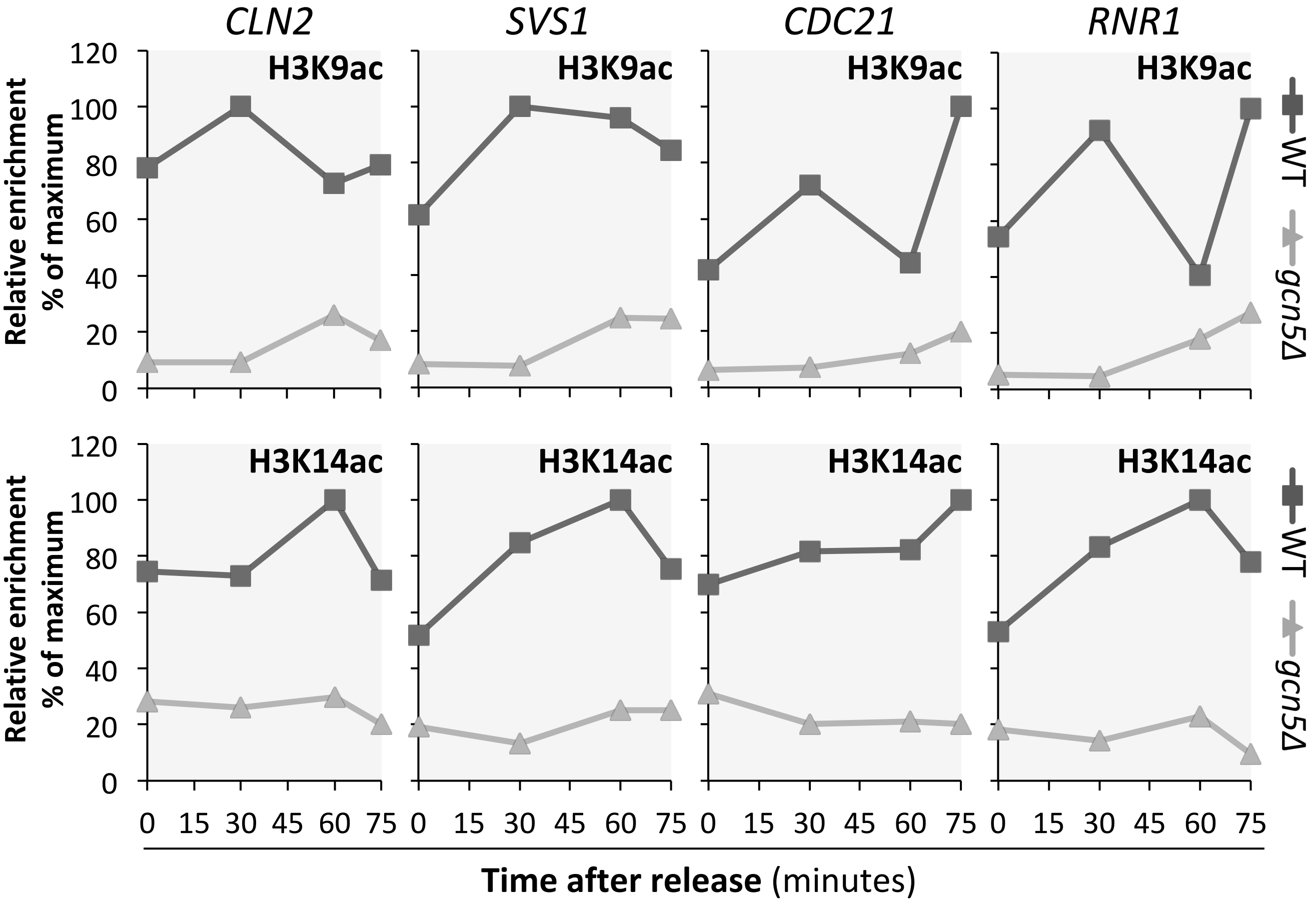

Supplementary Figure 4. Cells lacking Gcn5 have reduced H3K9 and H3K14 acetylation in synchronous cultures. α-factor cell cycle arrest of wild-type and *gcn5Δ* cell cultures. Cells were collected at 0, 30, 60 and 75 minutes after release from α-factor and analysed by ChIP. The same amount of *S. pombe* culture was added upon fixation for normalisation. The signal was firstly normalised to total H3 ChIP and then to the signal at the *S. pombe ACT1* promoter, levels are presented relative to the maximum signal (100%).

Supplementary Figure-5 de Bruin

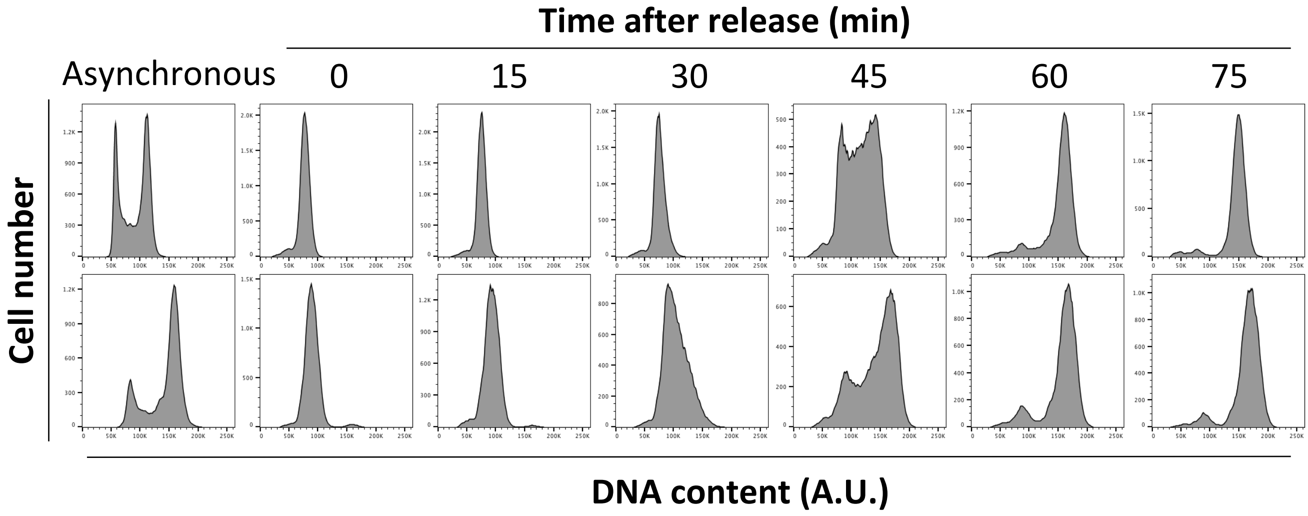

Supplementary Figure 5. Cells lacking Rpd3 enter S phase more quickly than wild-type cells. Flow cytometry analysis of wild-type and *rpd3Δ* cells during α-factor arrest and release. Both cultures are arrested well in G1 phase however *rpd3Δ* cells progress into S phase earlier than wild-type cells.
